# Supplementary material for: The Impact of a 14-Day Altitude Training Camp on Olympic-Level Open-Water Swimmers’ Sleep
Source: Int J Environ Res Public Health. 2022 Apr 2;19(7):4253. doi: 10.3390/ijerph19074253 (PMC8998594; doi:10.3390/ijerph19074253)
Supplement: Supplementary file 1 [file ijerph-19-04253-s001.zip › ijerph-1594228-supplementary.pdf]

Supplementary Table S1

		D-1 am		D-1 pm		D-2 am		D-2 pm		D-3 am		D-4 am		D-4 pm		D-5 am		D-5 pm		D-6 am	
		Volume (km)	sRPE	Volume (km)	sRPE	Volume (km)	sRPE	Volume (km)	sRPE	Volume (km)	sRPE	Volume (km)	sRPE	Volume (km)	sRPE	Volume (km)	sRPE	Volume (km)	sRPE	Volume (km)	sRPE
	Z1	6.80 ± 3.04		0.00 ± 0.00		6.70 ± 0.00		7.70 ± 0.00		7.60 ± 3.43		3.60 ± 0.00		6.10 ± 0.00		0.00 ± 0.00		0.00 ± 0.00		7.36 ± 3.29	
**PRE**	Z2	0.00 ± 0.00	3.06 ± 1.24	0.00 ± 0.00		0.00 ± 0.00	5.36 ± 2.24	0.00 ± 0.00	4.86 ± 1.83	0.74 ± 0.35	5.12 ± 2.86	4.20 ± 0.00	6.24 + 1.08	0.00 ± 0.00	7.66 + 0.73	0.00 ± 0.00		0.00 ± 0.00		1.28 ± 0.88	6.64 + 1.75
	Z3	0.00 ± 0.00		0.00 ± 0.00		0.30 ± 0.13		0.00 ± 0.00		0.24 ± 0.16		0.00 ± 0.00		0.90 ± 0.00		0.00 ± 0.00		0.00 ± 0.00		0.00 ± 0.00	
	Z1	7.20 ± 0.00		8.40 ± 0.00		6.20 ± 0.00		7.70 ± 0.00		8.20 ± 3.67		7.40 ± 0.00		6.40 ± 0.00		8.40 ± 0.00		5.80 ± 0.00		8.20 ± 3.67	
**T2**	Z2	0.00 ± 0.00	2.6 ± 1.69	0.00 ± 0.00	5.52 ± 1.12	0.00 ± 0.00	7.56 ± 1.11	0.60 ± 0.00	3.42 ± 1.92	1.60 ± 0.72	7.78 ± 0.29	1.20 ± 0.00	5.96 ± 0.33	0.20 ± 0.00	8.50 ± 1.51	0.00 ± 0.00	2.80 ± 1.29	0.00 ± 0.00	4.22 ± 2.23	0.90 ± 0.40	8.00 ± 1.44
	Z3	0.00 ± 0.00		0.00 ± 0.00		0.80 ± 0.0		0.60 ± 0.00		0.00 ± 0.00		0.00 ± 0.00		0.50 ± 0.00		0.00 ± 0.00		1.20 ± 0.00		0.90 ± 0.40	
	Z1	7.20 ± 0.00		7.86 ± 1.21		7.16 ± 0.54		7.94 ± 0.13		7.70 ± 3.44		4.80 ± 0.00		6.20 ± 0.00		7.70 ± 0.00		5.90 ± 0.00		6.85 ± 3.06	
**T3**	Z2	0.00 ± 0.00	4.34 ± 2.35	0.00 ± 0.00	5.88 ± 2.13	0.00 ± 0.00	3.30 ± 2.36	0.12 ± 0.27	3.30 ± 1.13	0.78 ± 0.42	8.54 ± 0.86	1.20 ± 0.00	3.56 ± 1.42	0.60 ± 0.00	7.42 + 1.47	0.00 ± 0.00	3.04 ± 1.21	0.60 ± 0.00	4.76 ± 2.44	1.00 ± 0.45	5.86 ± 0.69
	Z3	0.00 ± 0.00		0.36 ± 0.80		0.16 ± 0.36		0.12 ± 0.27		0.66 ± 0.85		1.20 ± 0.00		0.60 ± 0.00		0.00 ± 0.00		0.60 ± 0.00		0.15 ± 0.07	

The table shows the daily training volume (km) including sRPE in PRE and during the 2 weeks (i.e T2 and T3) of the training camp.
